# Caught in a downward spiral? The bidirectional relationship among food insecurity, financial strain, and depression and anxiety: A NESDA study

**DOI:** 10.1192/j.eurpsy.2026.12243

**Published:** 2026-07-20

**Authors:** Annemijne N. Nelissen, Stephanie E.E.C. Bauduin, Nienke J. de Bles, Nathaly Rius Ottenheim, Willemijn J. Maas, Brenda W.J.H. Penninx, Wessel A. van Eeden, Erik J. Giltay

**Affiliations:** 1 Department of Psychiatry & Psychology, Leiden University Medical Center, The Netherlands; 2 Academic Hospital Groningen: Universitair Medisch Centrum Groningen, Netherlands; 3 Amsterdam UMC VUMC Site: Amsterdam UMC Locatie VUmc, Netherlands

**Keywords:** anxiety, depression, financial strain, food insecurity, longitudinal studies

## Abstract

**Background:**

The relationship among food insecurity, financial strain, and depression and anxiety is complex and likely bidirectional, yet most studies have examined this relationship unidirectionally and cross-sectionally. Our study investigates the longitudinal bidirectional relationship among depression and/or anxiety and food insecurity and financial strain.

**Methods:**

Food insecurity and financial strain were measured at four waves across 13 years in the NESDA cohort. Clinical diagnoses of depression and anxiety were determined using the CIDI. Symptom severity was assessed using the IDS-SR and BAI. Lagged multilevel models examined whether food insecurity and financial strain predicted changes in depression and/or anxiety and vice versa. All models adjusted for age, sex, education, household income contributors, and prior outcome levels.

**Results:**

Among 2,567 individuals (mean age 42.4 years (SD 13.2), 34.6% women), higher food insecurity and financial strain significantly predicted increased odds of onset of depressive disorders (OR = 2.12, 95% CI: 1.43–3.15 and OR = 1.92, 95% CI: 1.51–2.45, respectively), and greater depressive symptom severity at the next wave (p’s for trend < .001 and p = .001, respectively). Associations with anxiety disorders and symptom severity were weaker. Conversely, depressive disorders and symptoms predicted higher subsequent food insecurity and financial strain, whereas anxiety symptoms did not.

**Conclusions:**

Our findings support a bidirectional temporal association among food insecurity, financial strain and depression, with weaker evidence for anxiety. Approaches that address financial hardship and mental health may be beneficial. Further long-term studies in diverse populations are needed to clarify mechanisms linking financial insecurity with mental health and to guide effective intervention development.

## Introduction

Global poverty is declining rapidly due to economic growth and development [1]. However, many people, including those in high-income nations, still struggle financially in daily life. In 2024, 21% of the European population were at risk of poverty or social exclusion [2], and approximately one third of Dutch households had difficulty covering expenses [3]. Financial insecurity may manifest in various forms, such as not having enough money to buy food or struggling to make ends meet financially by the end of the month [4]. Gaining insight into the relationship between financial insecurity and mental health may help to better understand the mechanisms that influence both.

Financial insecurity has been widely linked to poor mental health outcomes in the general population [5, 6]. A meta-analysis from 2020 including 372,143 participants from the general population, mostly from developed countries, found that food insecurity (the uncertainty of having enough money to buy enough food) was significantly correlated with a higher risk of depressive symptoms and stress, but not with anxiety [7]. A comprehensive review from 2022 covering 109 studies found the same associations among food insecurity and depression, stress, and anxiety, and also linked food insecurity with an increase in other mental health outcomes, such as child depression and child hyperactivity [8]. Furthermore, other forms of economic insecurity, such as job or housing insecurity, showed similar patterns [9, 10]. Together, these studies show that a range of subtypes of financial insecurity are associated with increased depression and anxiety symptoms.

Most of the aforementioned studies have only investigated the effects of financial insecurity on mental health and not vice versa, and have been limited to cross-sectional analyses, lacking the temporal perspective to examine the dynamic relationship between these factors over time, as well as the potential causal relationship between the two. Only two of the studies conducted in this field analyzed the temporal effect of food insecurity on depression, and found that food insecurity predicted depression at later time points [11, 12]. These studies had follow-up periods of 8 years and 18 weeks and included 20,415 and 776 participants, respectively.

Literature on the reverse effect, the extent to which depression and anxiety may contribute to financial insecurity, is relatively sparse. The limited longitudinal research available suggests that depressive symptoms predicted later financial insecurity [13–15]. These studies used prospective designs with follow-up periods of two to three years. The latter two studies also examined the bidirectional associations and found a bidirectional association between financial insecurity and maternal depression. However, these studies focused on specific populations, such as low-income households or women only, and relied on symptom-based screening tools, such as the Center for Epidemiologic Studies Depression Scale (CES-D) or Composite International Diagnostic Interview Short-Form (CIDI-SF). Overall, there have been limited studies investigating the longitudinal relationship between clinically diagnosed depression and anxiety and financial insecurity. Thus, despite growing evidence linking financial insecurity to mental health problems, the temporal and potentially bidirectional relationship remains understudied.

A previous analysis within the current NESDA cohort investigated whether financial strain predicted the onset or recurrence of depressive or anxiety disorders at 4 year follow-up [16]. It found that financial strain was associated with having a current depressive or anxiety disorder at baseline, but did not predict onset or recurrence of these disorders over time. Our study extends these findings as it examined bidirectional associations over 13 years across four waves, using diagnoses based on the Composite International Diagnostic Interview (CIDI). In sum, this study aims to examine the bidirectional link among financial insecurity and depression and/or anxiety diagnoses and symptom severity in participants with current, remitted, comorbid, or no diagnosis (i.e., healthy controls). In this study, financial insecurity is used as an overarching concept, operationalized by two indicators: food insecurity and financial strain. Based on the literature, we hypothesize that depression and financial insecurity will exhibit a bidirectional temporal relationship, which is expected to be stronger than the corresponding relationship with anxiety.

## Methods

### Study population

**Figure 1.**
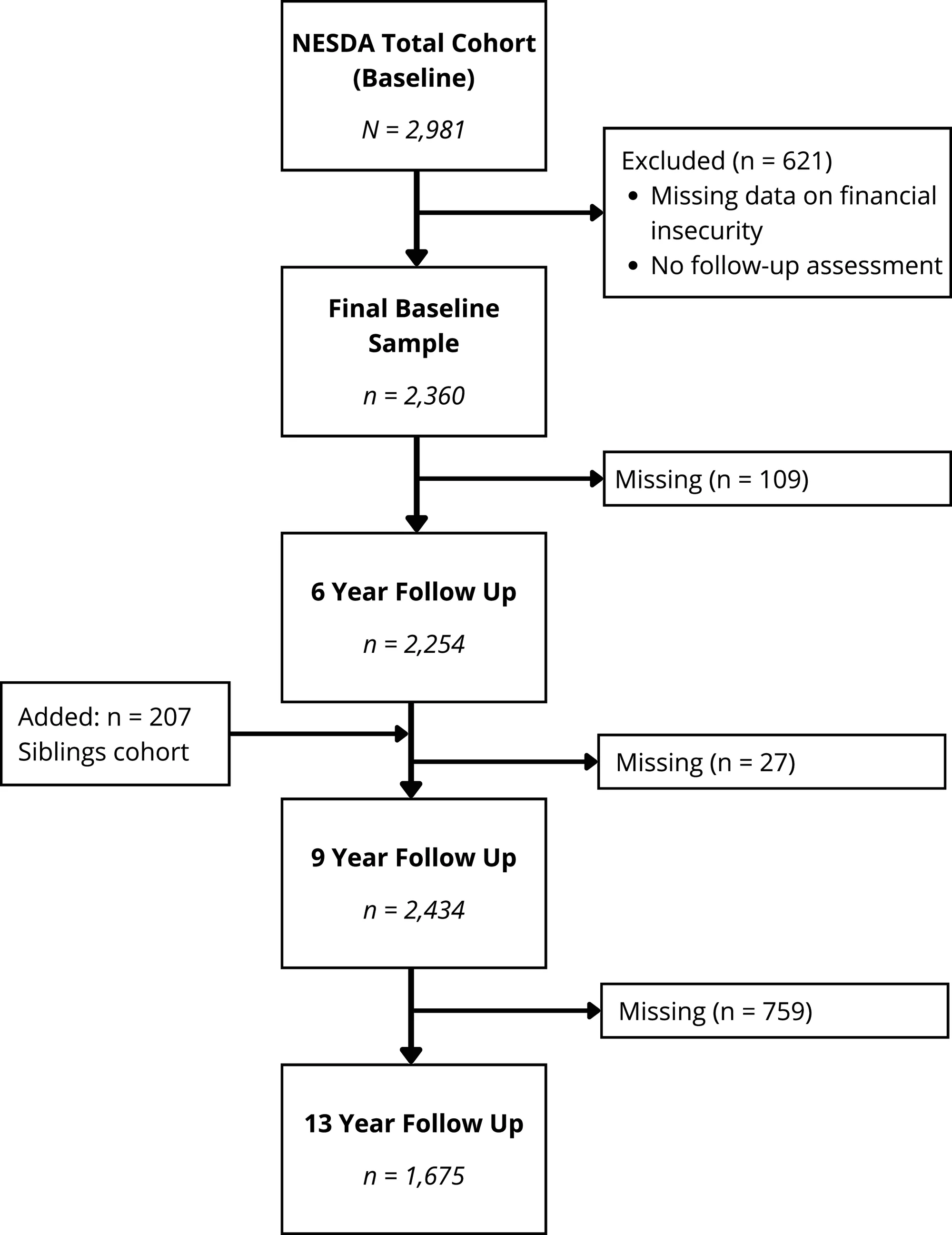
Flow diagram of the NESDA study sample and longitudinal follow-up. NESDA = Netherlands Study of Depression and Anxiety. The final analytical sample included n = 2,360 participants from the original cohort with complete data on financial insecurity and at least one follow-up assessment. At 9-year follow-up, a sibling cohort (n = 207) was added to the analytical sample.

Data were derived from the Netherlands Study of Depression and Anxiety (NESDA), an ongoing longitudinal, multi-site cohort study [17]. NESDA was designed to examine the long-term course, causes, and consequences of depression and anxiety disorders in the Dutch population since 2004 [17]. The study includes participants aged 18–65 years, with and without depression and/or anxiety symptoms, recruited from community, primary care, and specialized mental health care settings. The NESDA protocol was approved by the ethical committee of the participating research centers and written informed consent was obtained from all participants. For the purposes of this study, we analyzed existing data from baseline, 6, 9, and 13 years of follow-up (NESDA waves 1, 5, 6, and 7, respectively), as financial insecurity was not assessed in waves 2, 3, and 4. At baseline, a total of 2,360 participants from the original cohort who completed questionnaires on financial insecurity and had at least one complete follow-up assessment were included in the analysis. At 6-year follow-up (wave 5), a total of 2,254 participants (95.4%) remained. At 9-year follow-up (wave 6), a NESDA sub-study involving siblings of NESDA participants was introduced. Of the 367 available siblings, we applied the same exclusion criteria (i.e., requiring complete financial insecurity data and at least one assessment), resulting in 207 siblings being added to the analytical sample. The total sample included 2,434 participants, reflecting retention of the core cohort as well as the addition of 207 new participants [17]. The 13-year follow-up (wave 7) included a total of 1,675 participants. A detailed flow diagram of participant inclusion, exclusion, and longitudinal follow-up is provided in Figure 1.

### Measures

#### Food insecurity and financial strain

Financial insecurity was assessed using two indicators: food insecurity and financial strain. Both were assessed at baseline, 6-, 9-, and 13-year follow-up. Food insecurity was measured with the item: “How often do you find yourself without enough money to buy the food you and your family need?,” rated on a 4-point Likert scale (1 = never; 2 = occasionally; 3 = regularly; 4 = often). Financial strain was measured with the item: “In general, how is your financial situation at the end of the month?,” with response options: 1 = usually have money left over; 2 = just enough to get by; 3 = not enough to get by. For the descriptive analyses presented in Table 1, participants were categorized as financially insecure if they scored ≥2 on either item, or financially secure if they scored 1 on both. For longitudinal regression analyses, the two indicators (food insecurity and financial strain) were analyzed separately. Response categories were collapsed for food insecurity (i.e., scores of 3 and 4 were combined into a single category “regularly/often”) in all models examining financial insecurity items as predictor variables. When used as outcomes, the original 4-point ordinal scale was retained.

**Table 1.** Baseline sociodemographic and clinical factors according to financial insecurity

		Financial insecurity category		
	Baseline *N* = 2,567	Secure *N* = 1,327	Not secure *N* = 1,240	*p-value*
*Sociodemographic factors*				
Age in years, mean (SD)	42.4 (13.2)	44.5 (13.3)	40.3 (12.8)	<.001
Sex, *N* female (%)	888 (34.6)	531 (40.0)	357 (28.8)	<.001
Education in years, mean (SD)	12.5 (3.2)	13.0 (3.2)	11.9 (3.2)	<.001
*Financial characteristics*				
Household income contributors, *N* ≥ 2 (%)	1419 (55.4)	838 (63.2)	581 (46.9)	<.001
Net monthly household income, *N* (%)				<.001
Low (< €1800)	943 (37.2)	281 (22.1)	654 (53.3)	
Below average (€1800–2800)	761 (30.0)	409 (31.3)	352 (28.7)	
Average (€2801–3800)	568 (22.4)	395 (30.2)	173 (14.1)	
Above average (€3801–5000)	170 (6.7)	131 (10.0)	39 (3.2)	
High (> €5000)	92 (3.6)	83 (6.4)	9 (0.7)	
*Clinical characteristics*				
IDS-SR score, mean (SD)	19.6 (13.6)	17.5 (12.8)	21.9 (14.1)	<.001
BAI score, mean (SD)	10.8 (9.9)	9.5 (9.3)	12.1 (10.3)	<.001
Depression diagnosis (CIDI), *N* (%)	1605 (62.5)	732 (55.2)	873 (70.4)	<.001
Anxiety diagnosis (CIDI), *N* (%)	982 (38.3)	436 (32.9)	546 (44.0)	<.001
*Sample characteristics*				
Siblings, *N* (%)	207 (8.1)	132 (9.9)	75 (6.0)	<.001

*SD – standard deviation, IDS-SR – Inventory of Depressive Symptomatology (Self Report), BAI – Beck Anxiety Inventory; CIDI – Composite International Diagnostic Interview.*

*Note:* The descriptive baseline sample comprises 2,360 NESDA baseline participants plus 207 siblings whose baseline assessment occurred at wave 6. Group differences were tested using independent samples *t*-tests for continuous variables and chi-square tests for categorical variables. *P*-values indicate differences between financially secure and insecure participants. For the siblings subsample, values reflect their baseline assessment conducted at a separate wave.

#### Depression and anxiety diagnoses

Clinical diagnoses of depression (Major Depressive Disorder and dysthymia) and anxiety (Generalized Anxiety Disorder, panic disorder with or without agoraphobia, and social phobia) as defined by the DSM-IV classification system were identified using the CIDI [18] at baseline and the three follow-up waves. The presence of a current depressive or anxiety disorder was defined as an active diagnosis of any depressive and/or anxiety disorder in the past 6 months.

#### Depression and anxiety symptoms

Depressive symptoms were measured at all time points using the 28-item Inventory of Depressive Symptomatology Self Report (IDS-SR) [19], consisting of 28 items covering all symptoms of a depressive disorder according to the DSM-IV, rated on a 4-point Likert scale. The IDS-SR is a well-validated instrument with a high internal consistency (Cronbach’s α ≈ 0.92–0.94), good construct validity, and sensitivity to change.

Anxiety symptoms were measured using the 21-item Beck Anxiety Inventory (BAI) [20], which measures 14 somatic and 7 cognitive items on a 4-point Likert scale. The BAI has demonstrated high internal consistency (Cronbach’s α ≈ 0.92), good test–retest reliability, and strong discriminant validity.

#### Covariates

All models were adjusted for age, sex, and education in years, as these sociodemographic factors are associated with both food insecurity and financial strain, and with depression and anxiety. In addition, we adjusted for the number of household members who contributed to the household income. Finally, in the fully adjusted model, depression and anxiety were mutually adjusted for each other.

### Statistical analysis

Baseline sociodemographic and clinical characteristics were compared between financially secure and insecure participants using chi-square tests for categorical variables and independent-samples *t*-tests for continuous variables. To assess the stability of financial insecurity across the 13-year follow-up, we calculated intraclass correlation coefficients (ICCs) based on a two-way mixed-effects model with single measurements and absolute agreement, in which the repeated assessments (waves) were treated as fixed and the participants as random effects.

We used lagged multilevel models with repeated measures across four time points (baseline, 6-, 9-, and 13-year follow-up) to examine whether variable *X* at time *t* predicted changes in outcome *Y* at time *t* + 1, adjusting for the prior level of *Y.* Thus, multivariable multilevel logistic and linear regression analyses were performed to examine potential relationships among food insecurity, financial strain and depression and/or anxiety over time. Binary outcomes (e.g., presence of depressive/anxiety disorder) were analyzed using multilevel logistic regression, and continuous outcomes (e.g., symptom severity) using multilevel linear regression. All models included a random intercept for participants to account for within-person clustering over time and were adjusted for age, sex, educational level, and the time-varying variable for the number of household income contributors. Additionally, in models where depression or anxiety severity served as predictors, these were mutually adjusted for the related predictor variables to isolate the unique association, beyond shared variance with related constructs. Specifically, when examining depression as a predictor, we adjusted for concurrent anxiety symptoms, and vice versa. Variables were standardized (z-scores) for visualization in the forest plots and to facilitate direct comparison of effect sizes across different outcomes. We estimated adjusted marginal risk differences, defined as the average model-based differences in predicted probabilities between the three categories of either food insecurity or financial strain, with 95% confidence intervals, for the risk of depressive and anxiety disorders. Because follow-up intervals among waves differed in length (6, 3, and 4 years), separate lagged models were also estimated for each interval (baseline–6 years, 6–9 years, and 9–13 years).

Food insecurity and financial strain were analyzed as both an ordinal item-level variable and as a dichotomous variable (financially secure versus insecure). Results are presented as standardized beta coefficients or odds ratios (per standard deviation increase) with 95% confidence intervals.

In our mixed models, all available observations per person were included, dropping only rows with missing values, which yields valid estimates under the assumption of Missing at Random (MAR).

To test the robustness of our findings, we performed several sensitivity analyses. First, the longitudinal analyses were repeated with fewer timepoints that were closer together in time by excluding the baseline, providing more comparable lag intervals and reducing potential bias from baseline oversampling of current disorders. Second, to evaluate whether the associations among food insecurity, financial strain and depression and/or anxiety were independent of objective income level, we conducted analyses limited to participants with household income ≤ €2800. Observations were restricted to each of the respective time points at which participants reported a household income ≤ €2800, accounting for time-varying income levels. Third, we examined cross-sectional associations at wave 6 between financial insecurity and depressive and anxiety disorders, symptoms, and worry (Penn State Worry Questionnaire (PSWQ)), using the same model specifications and covariate adjustments. Binary outcomes (e.g., depressive or anxiety disorders) were analyzed using logistic regression analyses, and continuous outcomes (symptom severity) were analyzed using linear regression analyses.

All analyses were conducted in R version 4.4.3, using the main packages “lme4”(version 1.1–37), “lmerTest”(version 3.1–3), and “forestplot”(version 3.1.3).

## Results

### Sociodemographic characteristics at baseline

Table 1 shows the sociodemographic, financial, and clinical characteristics of the study sample at baseline. The baseline sample consisted of 2,567 individuals: 2,360 participants assessed at the original NESDA baseline (wave 1) and 207 siblings whose first NESDA assessment occurred at wave 6. Of these, 1,327 (51.7%) were categorized as financially secure and 1,240 (48.3%) as financially insecure. Participants had a mean age of 42.4 years (SD 13.2), and 34.6% were women. Statistically significant group differences were observed across all reported characteristics (all p-values <0.01). The financially insecure group had, among other differences, fewer years of education, fewer household income contributors, and lower household income.

### Prevalence of financial strain and food insecurity over time

**Figure 2.**
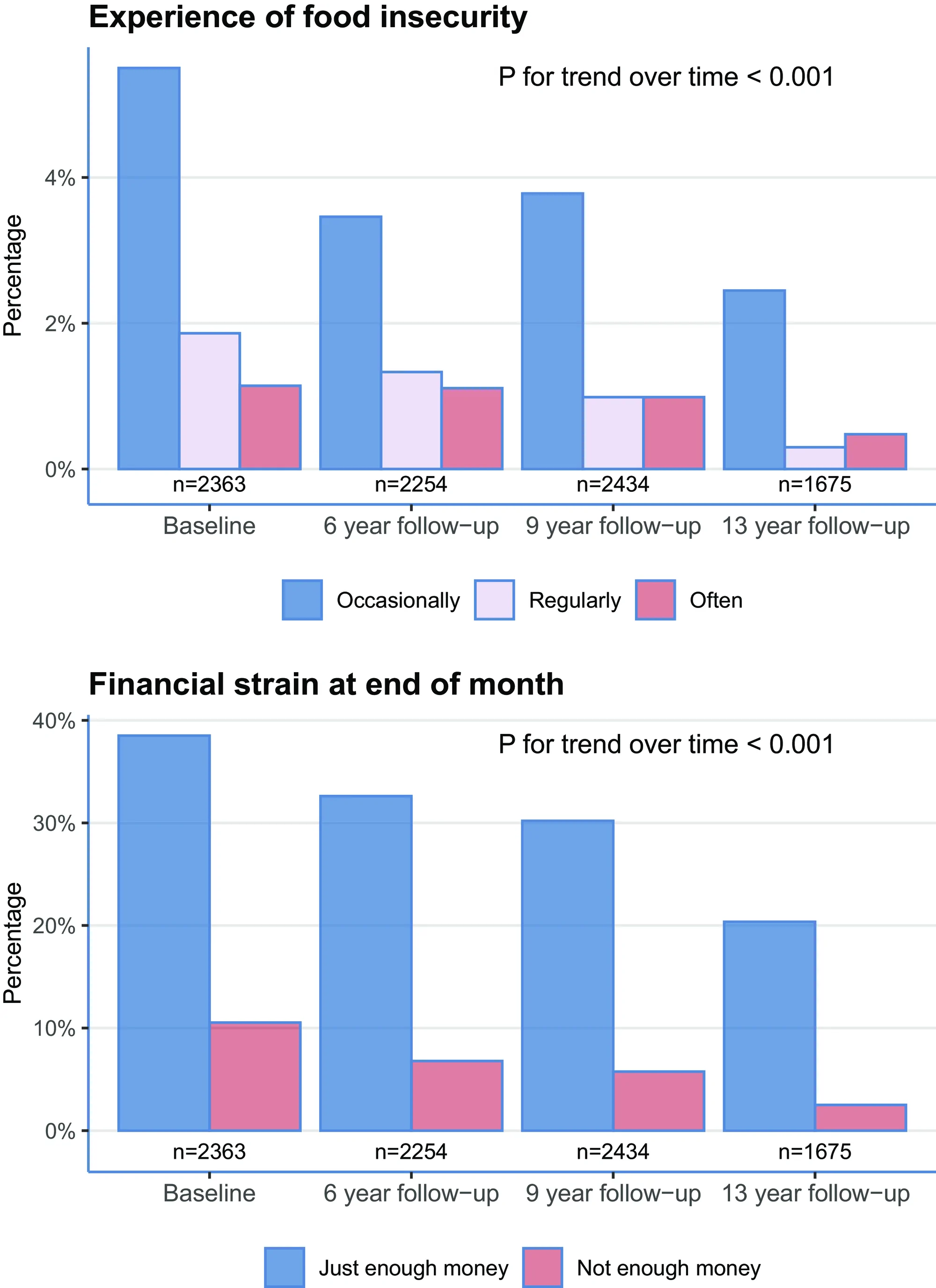
Prevalence of food insecurity over time and prevalence of financial strain over time.

The prevalence of both food insecurity and financial strain declined significantly over the 13-year follow-up period (*P* for trend <0.001 for both items; Figure 2). At baseline, 5% of participants reported occasionally lacking money to buy food, while 2 and 1% reported experiencing this regularly and often, respectively. These figures decreased steadily, with only 0.5% reporting frequent food insecurity after 13 years. A similar decline was observed for financial strain, with the proportion reporting not having enough money at the end of the month decreasing from 11% to 2%.

### Food insecurity and financial strain as predictors of depression or anxiety disorders at the next wave

ICCs indicated modest stability over 13 years of follow-up, with 34% of the variance in financial strain and 39% in food insecurity reflecting stable between-person differences, and the remainder reflecting within-person change across the 13-year follow-up.

**Figure 3.**
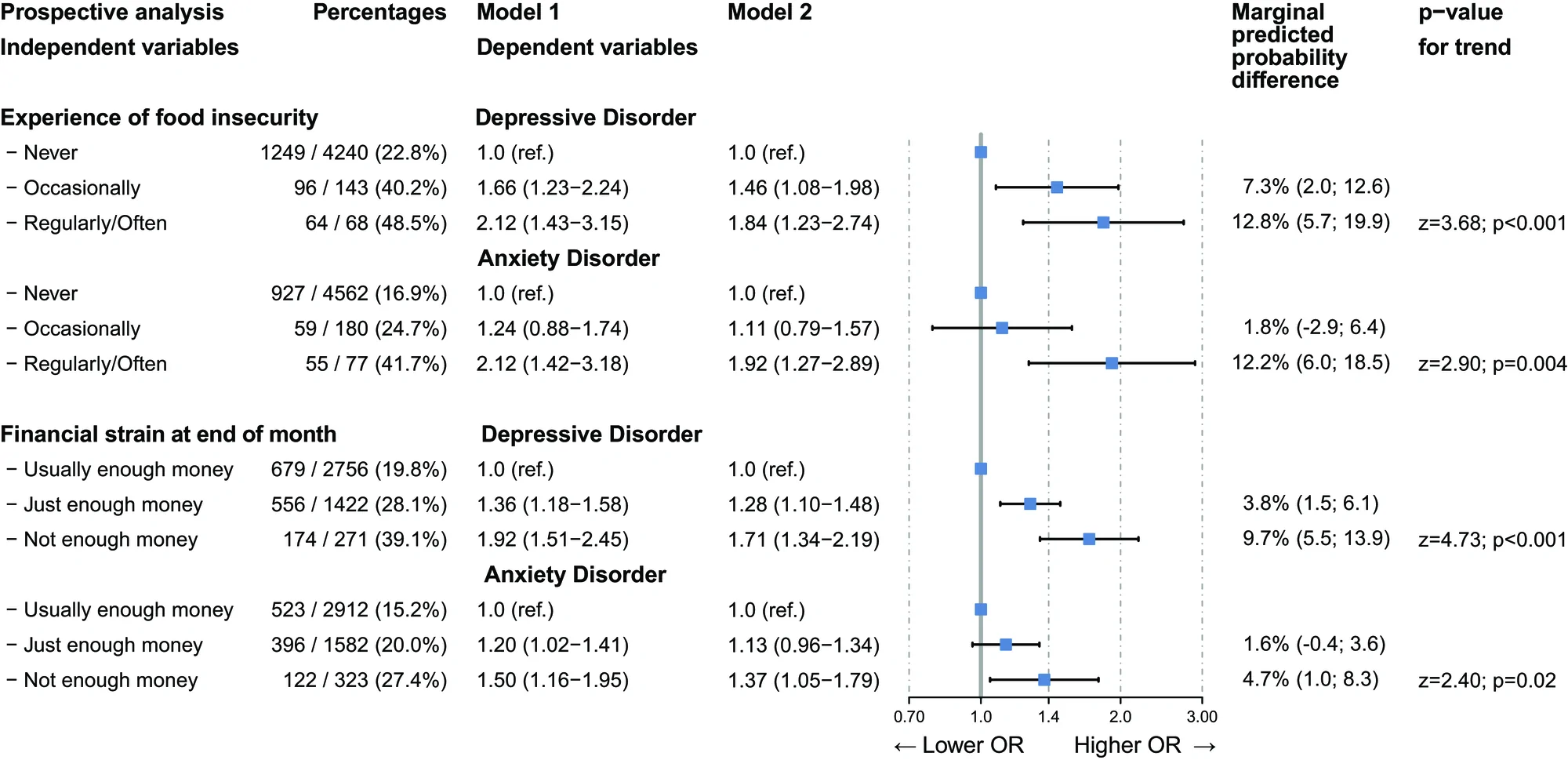
Prospective lagged multilevel logistic regression analysis: food insecurity and financial strain predicting depression and anxiety disorder at the next wave over a 13-year follow-up. Odds ratios (ORs) and marginal predicted probability differences and 95% confidence intervals (CIs) (in parentheses) for current depressive and anxiety disorders (defined as an active diagnosis of any depressive or anxiety disorder in the past 6 months) across categories of food insecurity and financial strain. Estimated values were derived from prospective lagged multilevel logistic regression analyses including repeated measures across four waves over 13 years. Percentages indicate the proportion of participants within each financial insecurity category. Model 2 adjusted for age, sex, education, number of household income contributors, and comorbid baseline diagnosis (i.e., anxiety disorder in models predicting depressive disorder, and vice versa). The right-hand plots show estimated ORs (with 95% CIs). Significance of the linear trends across categories was tested using z-statistics.

Participants who reported food insecurity or financial strain had significantly higher odds of developing depressive and anxiety disorders at the next assessment wave across the 13-year follow-up (Figure 3). Individuals reporting regular/often food insecurity had a 12.8% and 12.2% higher marginal predicted probability of depressive and anxiety disorder, respectively, compared to those who reported never experiencing food insecurity, with smaller but consistent increases observed for financial strain (9.7% and 4.7%, respectively).

In the adjusted model, both occasional and regular/often food insecurity were significantly associated with higher odds of depressive disorder at the next wave (OR = 1.46, 95% CI: 1.08–1.98; OR = 1.84, 95% CI: 1.23–2.75, respectively). A significant linear trend across categories indicated a dose–response relationship (*z* = 3.68, *p* < 0.001). Food insecurity also predicted subsequent anxiety disorder, although associations were weaker. Only the highest category had a significantly increased risk of anxiety disorder (OR = 1.92, 95% CI: 1.27–2.89). Although not all odds ratios reached statistical significance, the overall linear trend across categories was significant (*z* = 2.90, *p* = 0.004), suggesting a gradual increase in risk with higher levels of food insecurity.

Perceived financial strain at the end of the month was likewise associated with higher odds of depressive disorder at the next wave. Participants who reported not having enough money at the end of the month had significantly increased odds of depressive disorder (OR = 1.71, 95% CI: 1.34–2.19), and those reporting just enough money also showed elevated odds (OR = 1.28, 95% CI: 1.10–1.48). A strong linear trend was observed across categories (*z* = 4.73, *p* < 0.001). Associations between financial strain and anxiety disorders were weaker. Only participants who reported not having enough money at the end of the month had increased odds of an anxiety disorder at the next wave (OR = 1.37, 95% CI: 1.05–1.79). A linear trend was observed across categories (z = 2.40, *p* = 0.02).

### Food insecurity and financial strain as predictors of depression or anxiety symptom severity at the next wave

**Figure 4.**
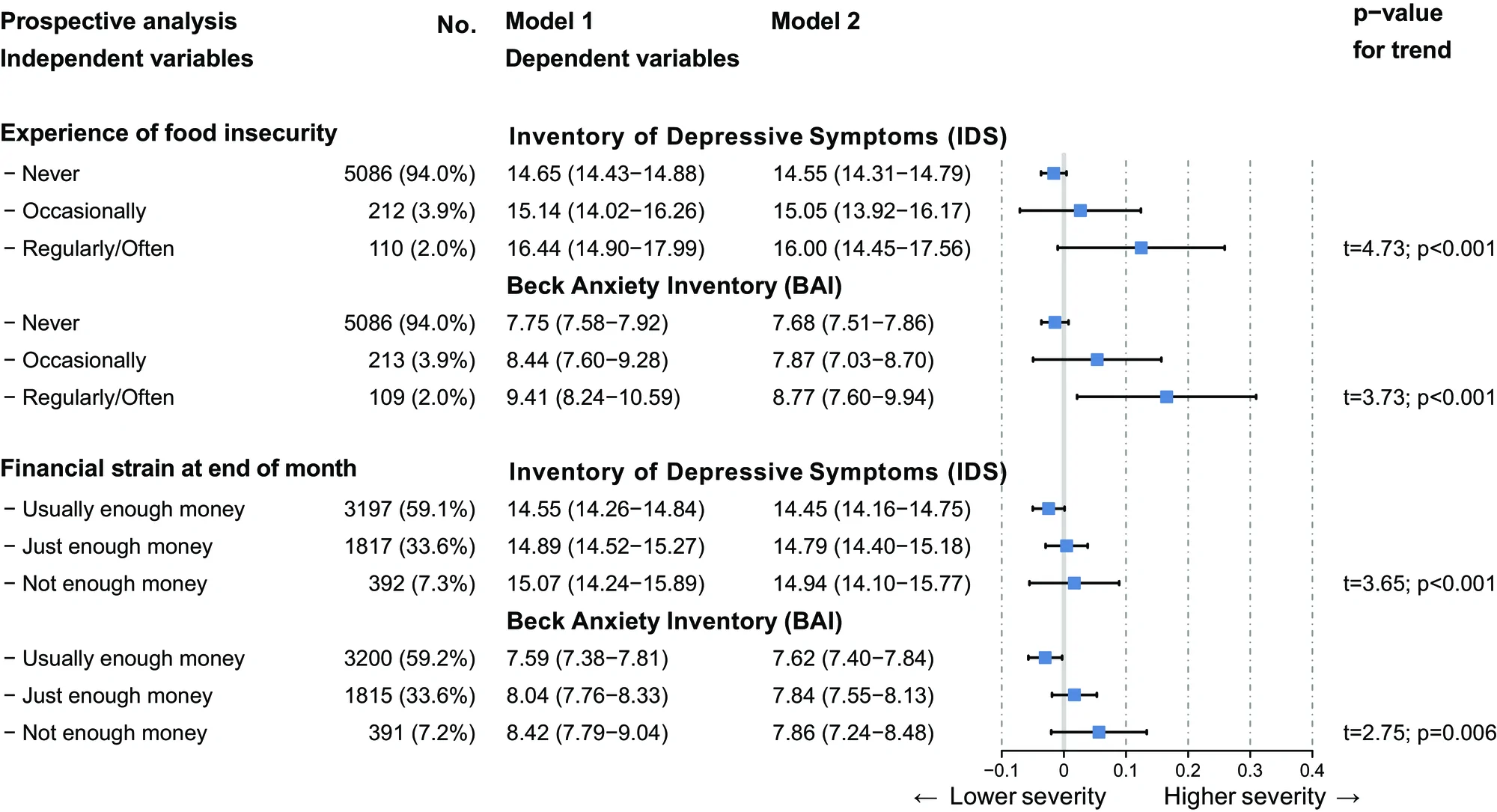
Prospective lagged multilevel linear regression analysis: food insecurity and financial strain predicting depressive and anxiety symptom severity at the next wave over a 13-year follow-up. Mean scores and 95% confidence intervals (CIs) (in parentheses) for depressive (IDS-SR) and anxiety (BAI) symptom severity across categories of food insecurity and financial strain. Estimated values were derived from prospective lagged multilevel linear regression analyses including repeated measures across 4 waves over 13 years. Percentages indicate the proportion of participants within each financial insecurity category. Model 2 adjusted for age, sex, education, number of household income contributors and comorbid baseline symptoms (i.e., BAI in models predicting IDS and vice versa). The right-hand plots show standardized beta-coefficients, representing standardized mean differences in symptom severity across financial insecurity categories. Significance of the linear trends across categories were tested using t-statistics.

Participants reporting greater food insecurity and financial strain were more likely to have elevated symptom levels of depression and/or anxiety at the next wave (Figure 4).

The small subgroup of participants (2%) reporting regular or frequent food insecurity showed the highest mean levels of both depressive and anxiety symptoms, representing an increase of approximately 1.5 IDS points and 1.1 BAI points compared to those reporting no food insecurity. Participants reporting the highest level of financial strain (7.3%) also had elevated symptom levels, though to a lesser extent. Significant linear trends were observed for both indicators of financial insecurity, indicating that higher levels of food insecurity and financial strain were associated with greater depressive symptom severity at the next wave (IDS-SR: *t* = 4.73, *p* < 0.001; *t* = 3.65, *p* < 0.001, respectively) and greater anxiety symptom severity at the next wave (BAI: *t* = 3.73, *p* < 0.001; *t* = 2.75, *p* = 0.006, respectively).

### Depression and anxiety diagnoses and symptom severity as predictors of food insecurity and financial strain at the next wave

**Figure 5.**
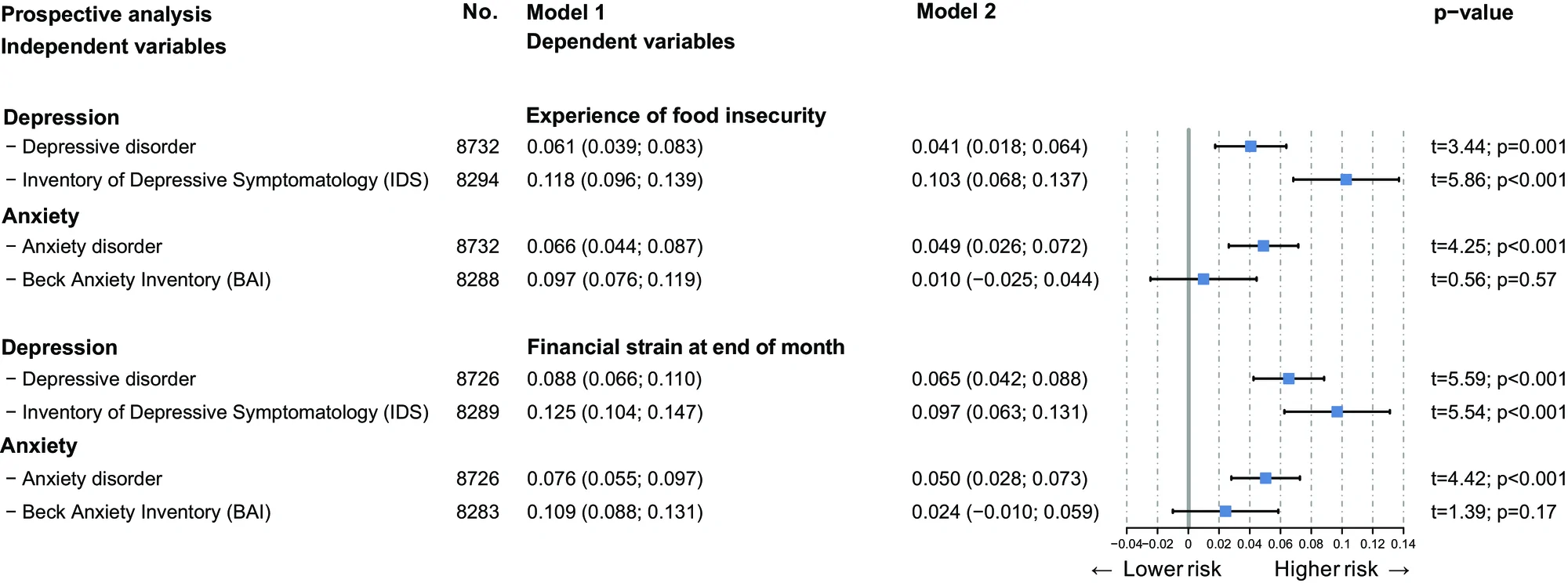
Prospective lagged multilevel linear regression analysis: depression and anxiety predicting food insecurity and financial strain at the next wave over a 13-year follow-up. Standardized regression coefficients (β) and 95% confidence intervals (CIs) (in parentheses) for food insecurity and financial strain outcomes. Estimated values were derived from prospective lagged multilevel linear regression analyses including repeated measures across 4 waves over 13 years. Model 2 adjusted for age, sex, education, number of household income contributors, and comorbid baseline diagnoses and symptoms. The right-hand plots show standardized β-coefficients, representing associations between depressive and anxiety disorders and symptoms and subsequent levels of financial insecurity. Significance of these regression coefficients was tested using t-statistics.

Depression and anxiety were prospectively associated with increased levels of food insecurity and financial strain at the next wave across the 13-year follow-up (Figure 5). Both depressive disorder (*β* = 0.04, 95% CI: 0.02–0.06; *p* = 0.001) and depressive symptoms (*β* = 0.10, 95% CI: 0.07–0.14; *p* < 0.001) were significantly associated with increased food insecurity. Similar associations were observed for financial strain (*β* = 0.07, 95% CI 0.04–0.09; *p* < 0.001; and *β* = 0.10, 95% CI 0.07–0.13; *p* < 0.001, respectively). Anxiety disorder also predicted increased food insecurity and financial strain (*β* = 0.05, 95% CI 0.03–0.07; *p* < 0.001; and *β* = 0.05, 95% CI 0.03–0.07; *p* < 0.001, respectively), whereas anxiety symptoms did not.

### Sensitivity and cross-sectional analyses

Several sensitivity analyses were conducted to assess the robustness of the primary findings. First, associations were broadly consistent across time intervals, with some attenuation and wider confidence intervals in shorter follow-up periods (Supplementary Tables S1–S3), supporting the overall robustness of the observed associations. Second, findings remained consistent in a restricted three-wave model that excluded the baseline assessment (Supplementary Figures S1–S3). Third, sensitivity analysis limited to participants with household income ≤ €2800 showed largely similar patterns to our main analyses (Supplementary Figures S4–S6). Finally, the cross-sectional associations among food insecurity, financial strain and depressive and anxiety disorders, symptoms, and worry at wave 6 showed that worry did not remain a significant correlate in the multivariate analysis, whereas depressive symptom severity did (Supplementary Figure S7).

## Discussion

This study provides longitudinal evidence consistent with a bidirectional association among food insecurity, financial strain and clinically diagnosed depression and anxiety over a 13-year period. Depression and anxiety were predictive of increased food insecurity and financial strain at subsequent assessments, while financial strain and food insecurity predicted the onset of depressive disorders and higher depressive symptom severity. Associations were generally stronger for depression than for anxiety, and stronger for food insecurity than financial strain.

Our prospective associations support longitudinal evidence linking financial insecurity to depression [7, 8, 11, 12, 21]. Our study builds on prior research by including clinical diagnoses, repeated assessments, and lagged modeling over a long follow-up, unlike earlier studies with symptom-based or cross-sectional designs. Several mechanisms may underlie these associations, including stress-related, psychosocial, and socio-economic pathways [22-25]. Remarkably, worry did not appear to be the primary driver of this association. Instead, depressive symptom severity remained the most consistent correlate of food insecurity and financial strain. We found dose–response effects indicating that greater severity of food insecurity and financial strain amplified depressive symptom severity [8]. Additionally, food insecurity showed stronger associations than perceived financial strain, consistent with prior work indicating that material deprivation more directly affects mental health [15, 26]. The association extended to the low-income subgroup, underscoring that perceived financial strain involves intertwined material and psychological dimensions [27]. These findings contrast somewhat with a previous NESDA analysis [16], suggesting that broader indicators of financial insecurity or a longer observational window may reveal associations not detected previously.

The reverse pathway whereby depression and anxiety predict subsequent financial insecurity has, to our knowledge, been examined more scarcely [13–15]. Our findings align well with earlier findings that depressive disorders and symptoms predict later food insecurity and financial strain, extending prior findings by incorporating longer follow-up and clinical diagnoses. Mental health problems may contribute to financial insecurity through several mechanisms, including reduced cognitive functioning, motivation, and decision-making capacity, which can impair work performance and economic outcomes [22]. Symptoms such as poor concentration and hopelessness further hinder occupational functioning, while stigma, discrimination, and increased health care costs reinforce a syndemic cycle of financial insecurity and mental illness shaped by structural inequalities [28–30]. Previous studies among low-income mothers showed that depression predicted later food insecurity [13, 15], and reviews suggest depression often precedes financial strain [21]. Together, these results reinforce the view that depression plays a key role in the downward spiral between mental health and financial hardship.

Evidence shows that programs that combine financial counseling or employment support with psychological components were successful in reducing depressive symptoms and, in some cases, financial strain [21]. Recognizing financial insecurity as a modifiable social determinant of mental health is important for both clinical practice and public health. At the individual level, clinicians can integrate exploring financial stress into mental health care or refer to social services when needed, as addressing such stressors can support recovery. For individuals experiencing financial insecurity, early identification of (emerging) depressive symptoms and timely access to mental health support are equally important. At the family or system level, programs can target households to improve budgeting skills, reduce intergenerational stress, and strengthen social support. At the municipal level, community services can offer coordinated access to debt assistance, employment programs, and mental health care. At the governmental level, policies addressing income security, affordable housing, and access to mental health services may help reduce the intertwined burden of financial hardship and poor mental health [21, 31, 32].

Strengths of this study include the large, clinically characterized sample, with a 13-year repeated-measures design. The use of lagged multilevel models provided a strong statistical basis to detect temporal associations. Second, psychiatric diagnoses were established using a standardized diagnostic instrument [18]. Nonetheless, several limitations also need to be acknowledged. First, financial insecurity was assessed using two single-item indicators (food insecurity and financial strain), which may not capture its full multidimensionality, as captured by the USDA Household Food Security Survey Module [21]. Although our measures did not capture all dimensions of financial insecurity, previous research suggests that food insecurity and financial strain frequently co-occur with broader forms of material hardship, including housing instability, missed bill payments, and debt, and may therefore reflect wider economic vulnerability [33, 34]. Future research should use validated, multidimensional measures of financial insecurity that incorporate a broader set of previously identified factors and examine mechanisms such as stress and decision-making pathways. Second, despite extensive covariate adjustment, residual confounding cannot be excluded. Finally, attrition over the long follow-up might have led to underestimation of associations, as the most financially insecure participants may have been more likely to drop out of the cohort.

In conclusion, our findings support a bidirectional temporal association among food insecurity and financial strain, and depression and anxiety across 13 years. Financial hardship appears to be both associated with subsequent depressive symptoms and influenced by prior depression, suggesting that improving financial stability can be important for effective mental health prevention and care. Integrating attention to financial difficulties within mental health services may help mitigate the interrelated challenges of financial insecurity and mental health.

Future research should evaluate the effectiveness of integrated interventions, which have shown promise in improving mental health outcomes [31, 32].

## Supporting information

10.1192/j.eurpsy.2026.12243.sm001 Nelissen et al. supplementary material Nelissen et al. supplementary material

## Supplementary material

The supplementary material for this article can be found at http://doi.org/10.1192/j.eurpsy.2026.12243.
