# Supplementary material for: Caught in a downward spiral? The bidirectional relationship among food insecurity, financial strain, and depression and anxiety: A NESDA study
Source: Eur Psychiatry. 2026 Jul 20;69(1):e82. doi: 10.1192/j.eurpsy.2026.12243 (PMC13735837; doi:10.1192/j.eurpsy.2026.12243)
Supplement: Supplementary file 1 [file S0924933826122433sup001.pdf]

Supplementary materials

This appendix has been provided by the authors to give readers additional information about their work.

Supplement to: A.N. Nelissen, S.E.E.C. Bauduin, N.J. de Bles, N. Rius-Ottenheim, W.J. Maas, B.W.J.H. Penninx, W.A. van Eeden, E.J. Giltay

Caught in a downward spiral? The bidirectional relationship between food insecurity, financial strain, and depression and anxiety: a NESDA study

Supplementary materials

Caught in a downward spiral? The bidirectional relationship between food insecurity, financial strain, and depression and anxiety: a NESDA study

Authors: A.N. Nelissen, S.E.E.C. Bauduin, N.J. de Bles, N. Rius-Ottenheim, W.J. Maas, B.W.J.H. Penninx, W.A. van Eeden, E.J. Giltay

Overview of supplementary content Page

Table S1	Food insecurity and financial strain predicting depressive and anxiety disorders (interval-specific model)	iii
Table S2	Food insecurity and financial strain predicting IDS and BAI symptom severity (interval-specific model)	iv
Table S3	Depression and anxiety predicting subsequent food insecurity and financial strain (interval-specific model)	v
Figure S1	Food insecurity and financial strain predicting depressive and anxiety disorders (3-wave model)	vi
Figure S2	Food insecurity and financial strain predicting IDS and BAI symptom severity (3-wave model)	vii
Figure S3	Depression and anxiety predicting subsequent food insecurity and financial strain (3-wave model)	viii
Figure S4	Food insecurity and financial strain predicting depressive and anxiety disorders (low income)	ix
Figure S5	Food insecurity and financial strain predicting IDS and BAI symptom severity (low income)	x
Figure S6	Depression and anxiety predicting subsequent food insecurity and financial strain (low income)	xi
Figure S7	Cross-sectional associations at wave 6	xii

Supplementary Table 1. Interval-specific prospective lagged multilevel logistic regression analysis:

Food insecurity and financial strain predicting depressive and anxiety disorders (baseline→6 years, 6→9 years, and 9→13 years)

Predictor & Category	Interval 1 (6y)	Interval 2 (3y)	Interval 3 (4y)	Combined
	OR (95% CI)	OR (95% CI)	OR (95% CI)	OR (95% CI)
**Dependent variable: Depressive Disorder**				
Food Insecurity				
- Never	1.0 (ref)	1.0 (ref)	1.0 (ref)	1.0 (ref)
- Occasionally	1.39 (0.93-2.09)	1.58 (0.87-2.88)	1.45 (0.76-2.77)	1.46 (1.08-1.98)
- Regularly/Often	1.60 (0.94-2.72)	1.93 (0.91-4.07)	1.97 (0.82-4.77)	1.84 (1.23-2.74)
Financial Strain				
- Usually enough	1.0 (ref)	1.0 (ref)	1.0 (ref)	1.0 (ref)
- Just enough	1.20 (0.96-1.50)	1.43 (1.09-1.88)	1.24 (0.94-1.65)	1.28 (1.10-1.48)
- Not enough	1.55 (1.11-2.15)	1.74 (1.08-2.79)	2.10 (1.25-3.54)	1.71 (1.34-2.19)
**Dependent variable: Anxiety Disorder**				
Food Insecurity				
- Never	1.0 (ref)	1.0 (ref)	1.0 (ref)	1.0 (ref)
- Occasionally	1.21 (0.77-1.92)	failed to converge	1.33 (0.04-48.5)	1.11 (0.79-1.57)
- Regularly/Often	2.04 (1.13-3.68)	failed to converge	2.96 (0.05-184)	1.92 (1.27-2.89)
Financial Strain				
- Usually enough	1.0 (ref)	1.0 (ref)	1.0 (ref)	1.0 (ref)
- Just enough	1.06 (0.83-1.36)	1.33 (0.36-5.00)	1.13 (0.22-5.92)	1.13 (0.96-1.34)
- Not enough	1.30 (0.90-1.87)	1.48 (0.15-1.44)	1.92 (0.11-32.3)	1.37 (1.05-1.79)

Note. Odds ratios (ORs) and 95% confidence intervals (CIs) (in parentheses) for current depressive and anxiety disorders (defined as an active diagnosis of any depressive or anxiety disorder in the past 6 months) across categories of food insecurity and financial strain. Estimated values were derived from prospective lagged multilevel logistic regression analyses including repeated measures across 4 waves with different time intervals (6, 3, and 4 years). Percentages indicate the proportion of participants within each financial insecurity category. Models are adjusted for age, sex, education, number of household income contributors, and comorbid baseline diagnosis (i.e., anxiety disorder in models predicting depressive disorder, and vice versa).

Supplementary Table 2. Interval specific prospective lagged multilevel linear regression analysis:

Food insecurity and financial strain predicting depressive and anxiety symptom severity (baseline→6 years, 6→9 years, and 9→13 years)

Independent variables:	Dependent variables:							
	Inventory of Depressive Symptoms (IDS)				Beck Anxiety Inventory (BAI)			
**Interval**	**Interval 1 (6y)**	**Interval 2 (3y)**	**Interval 3 (4y)**	**Combined**	**Interval 1 (6y)**	**Interval 2 (3y)**	**Interval 3 (4y)**	**Combined**
**Variable**	**B (SE)**	**B (SE)**	**B (SE)**	**B (SE)**	**B (SE)**	**B (SE)**	**B (SE)**	**B (SE)**
Food insecurity								
**- Never**	14.98 (14.56-15.39)	14.60 (14.23-14.98)	13.89 (13.48-14.31)	14.55 (14.31-14.79)	8.22 (7.91-8.54)	7.41 (7.13-7.70)	7.25 (6.95-7.54)	7.68 (7.51-7.86)
**- Occasionally**	15.29 (13.61-16.98)	13.69 (11.67-15.70)	16.85 (14.60-19.11)	15.05 (13.92-16.17)	8.47 (7.21-9.74)	7.07 (5.52-8.62)	8.04 (6.41-9.66)	7.87 (7.03-8.70)
**- Regularly/Often**	16.20 (13.76-18.63)	14.06 (11.51-16.62)	18.43 (15.23-21.63)	16.00 (14.45-17.56)	8.92 (7.07-10.77)	7.30 (5.33-9.27)	10.74 (8.44-13.04)	8.77 (7.60-9.94)
Financial strain								
**- Usually enough**	14.95 (14.41-15.49)	14.47 (14.01-14.93)	13.79 (13.30-14.29)	14.45 (14.16-14.75)	8.26 (7.86-8.67)	7.21 (6.86-7.56)	7.25 (6.89-7.61)	7.62 (7.40-7.84)
**- Just enough**	15.08 (14.44-15.72)	14.84 (14.19-15.48)	14.21 (13.48-14.93)	14.79 (14.40-15.18)	8.25 (7.77-8.73)	7.78 (7.28-8.27)	7.29 (6.76-7.81)	7.84 (7.55-8.13)
**- Not enough**	15.13 (13.88-16.38)	14.16 (12.72-15.61)	16.60 (14.84-18.36)	14.94 (14.10-15.77)	8.13 (7.19-9.07)	7.51 (6.39-8.62)	8.55 (7.27-9.82)	7.86 (7.24-8.48)

Note. Mean scores and 95% confidence intervals (CIs) (in parentheses) for depressive (IDS-SR) and anxiety (BAI) symptom severity across categories of food insecurity and financial strain. Estimated values were derived from prospective lagged multilevel linear regression analyses including repeated measures across 4 waves with different time intervals (6, 3, and 4 years). Percentages indicate the proportion of participants within each financial insecurity category. Models are adjusted for age, sex, education, number of household income contributors and comorbid baseline symptoms (i.e., BAI in models predicting IDS and vice versa).

Supplementary Table 3. Interval-specific prospective lagged multilevel linear regression analysis:

Depressive and anxiety disorders and symptom severity predicting food insecurity and financial strain (baseline→6 years, 6→9 years, and 9→13 years)

**Independent variables**	**Dependent variables**							
	**Food insecurity**				**Financial strain**			
**Interval**	**Interval 1 (6y)**	**Interval 2 (3y)**	**Interval 3 (4y)**	**Combined**	**Interval 1 (6y)**	**Interval 2 (3y)**	**Interval 3 (4y)**	**Combined**
**Variable**	**B (SE)**	**B (SE)**	**B (SE)**	**B (SE)**	**B (SE)**	**B (SE)**	**B (SE)**	**B (SE)**
Depression								
**- Depressive disorder**	0.047 (0.016; 0.077)	0.031 (0.002; 0.059)	0.029 (-0.009; 0.066)	0.041 (0.018; 0.064)	0.066 (0.037; 0.095)	0.043 (0.014; 0.073)	0.032 (-0.005; 0.070)	0.065 (0.042; 0.088)
**- IDS score**	0.106 (0.064; 0.149)	0.071 (0.029; 0.114)	0.106 (0.047; 0.164)	0.103 (0.068; 0.137)	0.112 (0.071; 0.152)	0.107 (0.063; 0.151)	0.072 (0.014; 0.131)	0.097 (0.063; 0.131)
Anxiety								
**- Anxiety disorder**	0.045 (0.017; 0.072)	0.036 (0.009; 0.064)	0.052 (0.013; 0.091)	0.049 (0.026; 0.072)	0.056 (0.030; 0.082)	0.038 (0.010; 0.067)	0.031 (-0.008; 0.070)	0.050 (0.028; 0.073)
**- BAI score**	0.024 (-0.017; 0.066)	-0.001 (-0.044; 0.041)	-0.042 (-0.102; 0.018)	0.010 (-0.025; 0.044)	0.011 (-0.029; 0.051)	-0.012 (-0.056; 0.032)	0.010 (-0.050; 0.070)	0.024 (-0.010; 0.059)

Note. Standardized regression coefficients (β) and 95% confidence intervals (CIs) (in parentheses) for financial insecurity outcomes (food insecurity and financial strain). Estimated values were derived from prospective lagged multilevel linear regression analyses including repeated measures across 4 waves with different time intervals (6, 3, and 4 years). Models are adjusted for age, sex, education, number of household income contributors, and comorbid baseline diagnoses and symptoms.

Supplementary Figure 1. Prospective lagged multilevel logistic regression analysis:

Food insecurity and financial strain predicting depression / anxiety disorder at the next wave over a 7 year follow-up

Prospective analysis<br>Independent variables	Percentages	Model 1<br>Dependent variables	Model 2	p−value<br>for trend
**Experience of food insecurity**		**Depression**		
− Never	353 / 930 (27.5%)	1.0 (ref.)	1.0 (ref.)	
− Occasionally	37 / 47 (44.0%)	1.63 (0.99−2.70)	1.49 (0.88−2.54)	
− Regularly/Often	30 / 26 (53.6%)	2.02 (1.10−3.71)	1.48 (0.77−2.83)	z=1.69; p=0.09
		**Anxiety**		
− Never	236 / 1047 (18.4%)	1.0 (ref.)	1.0 (ref.)	
− Occasionally	16 / 68 (19.0%)	0.77 (0.41−1.44)	0.68 (0.36−1.30)	
− Regularly/Often	24 / 32 (42.9%)	1.96 (1.04−3.69)	1.87 (0.97−3.60)	z=1.15; p=0.25
**Financial strain at end of month**		**Depression**		
− Usually enough money	160 / 523 (23.4%)	1.0 (ref.)	1.0 (ref.)	
− Just enough money	196 / 399 (32.9%)	1.29 (0.98−1.69)	1.30 (0.98−1.73)	
− Not enough money	64 / 81 (44.1%)	1.77 (1.16−2.68)	1.59 (1.01−2.50)	z=2.33; p=0.02
		**Anxiety**		
− Usually enough money	117 / 566 (17.1%)	1.0 (ref.)	1.0 (ref.)	
− Just enough money	119 / 476 (20.0%)	1.23 (0.90−1.69)	1.10 (0.80−1.53)	
− Not enough money	40 / 105 (27.6%)	1.39 (0.86−2.22)	1.22 (0.74−2.00)	z=0.85; p=0.40

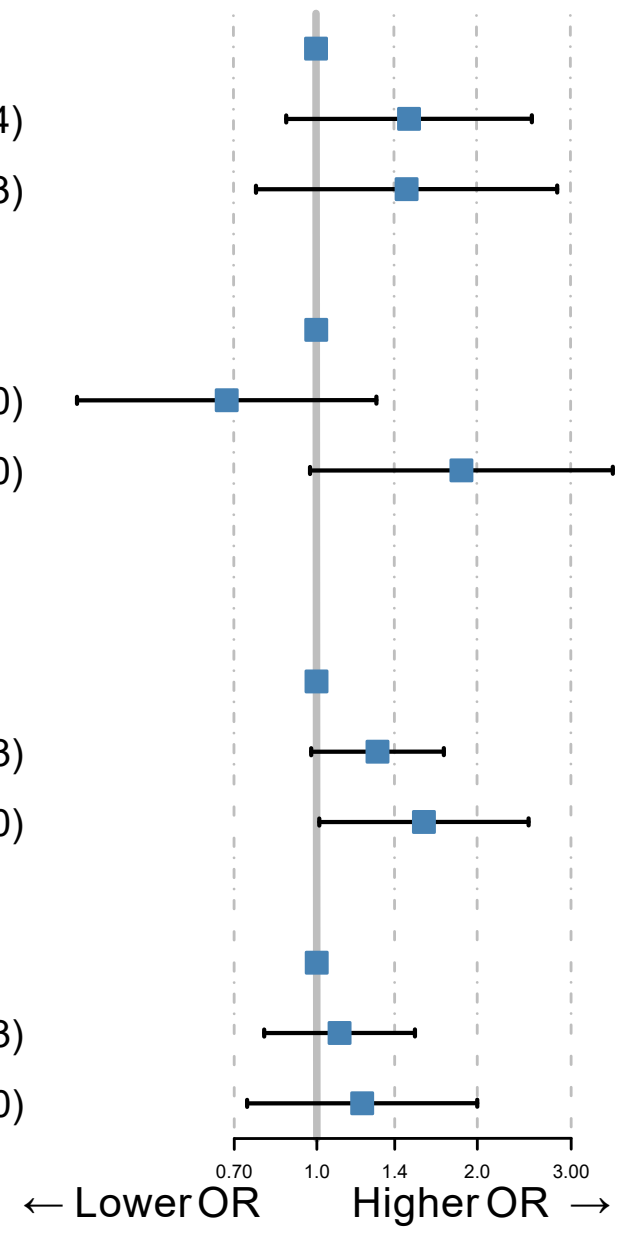

Note. Odds ratios (ORs) and 95% confidence intervals (CIs) (in parentheses) for current depressive and anxiety disorders (defined as an active diagnosis of any depressive or anxiety disorder in the past 6 months) across categories of food insecurity and financial strain. Estimated values were derived from prospective lagged multilevel logistic regression analyses including repeated measures across 4 waves over 7 years. Percentages indicate the proportion of participants within each financial insecurity category. Model 2 adjusted for age, sex, education, number of household income contributors, and comorbid baseline diagnosis (i.e., anxiety disorder in models predicting depressive disorder, and vice versa). The right-hand plots show estimated ORs (with 95% CIs). Significance of the linear trends across categories were tested using z-statistics.

Supplementary Figure 2. Prospective lagged multilevel linear regression analysis:

Food insecurity and financial strain predicting depressive and anxiety symptom severity at the next wave over a 7 year follow-up

**Prospective analysis**<br>**Independent variables**	**No.**	**Model 1**<br>**Dependent variables**	**Model 2**	**p−value**<br>**for trend**
**Experience of food insecurity**		**Inventory of Depressive Symptoms (IDS)**		
− Never	1152 (90.7%)	17.46 (16.99−17.94)	17.70 (17.18−18.23)	
− Occasionally	71 (5.6%)	17.36 (15.50−19.21)	16.82 (14.93−18.71)	
− Regularly/Often	47 (3.7%)	20.01 (17.72−22.29)	20.11 (17.80−22.42)	t=1.28; p=0.20
		**Beck Anxiety Inventory (BAI)**		
− Never	1151 (90.6%)	9.10 (8.73−9.47)	9.17 (8.76−9.58)	
− Occasionally	72 (5.7%)	9.18 (7.75−10.62)	8.50 (7.03−9.97)	
− Regularly/Often	47 (3.7%)	11.73 (9.94−13.52)	11.48 (9.68−13.28)	t=1.68; p=0.09
**Financial strain at end of month**		**Inventory of Depressive Symptoms (IDS)**		
− Usually enough money	619 (48.7%)	17.63 (16.98−18.27)	17.77 (17.09−18.45)	
− Just enough money	527 (41.5%)	17.41 (16.72−18.10)	17.64 (16.91−18.38)	
− Not enough money	124 (9.8%)	17.80 (16.40−19.20)	18.06 (16.58−19.53)	t=0.11; p=0.92
		**Beck Anxiety Inventory (BAI)**		
− Usually enough money	619 (48.7%)	8.89 (8.39−9.39)	9.07 (8.54−9.60)	
− Just enough money	527 (41.5%)	9.31 (8.77−9.84)	9.22 (8.65−9.80)	
− Not enough money	124 (9.8%)	10.26 (9.16−11.35)	10.01 (8.85−11.17)	t=1.26; p=0.21

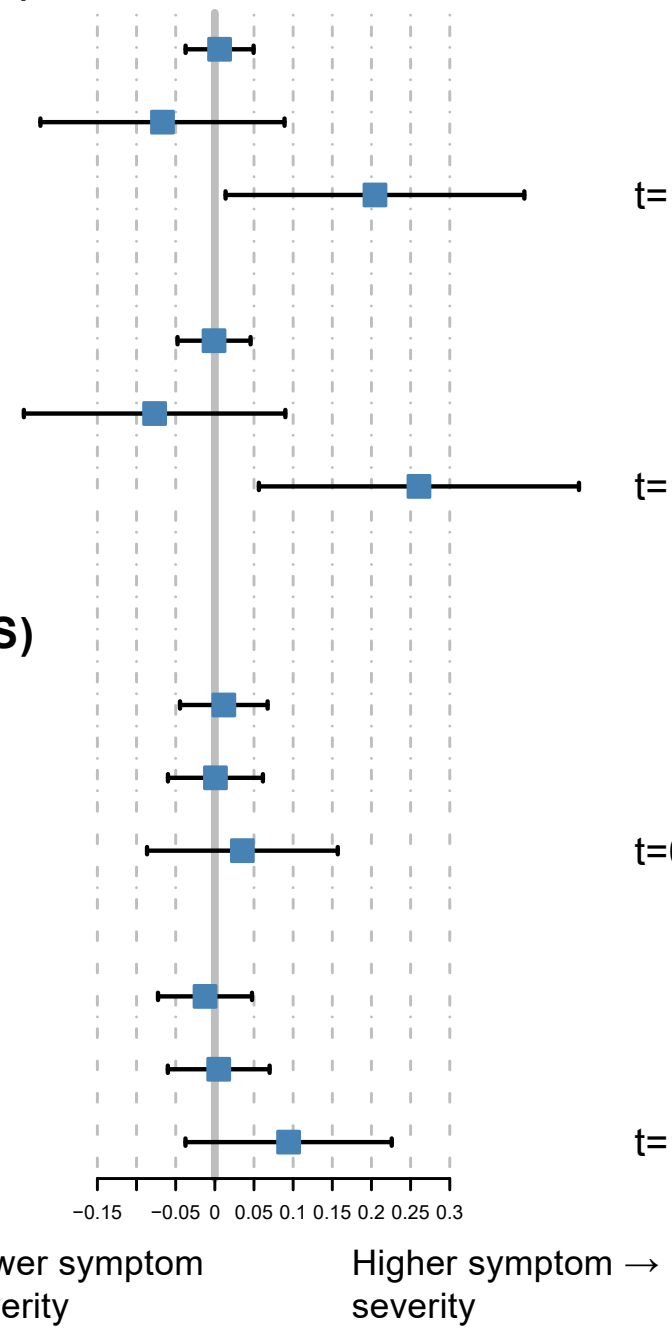

Note. Mean scores and 95% confidence intervals (CIs) (in parentheses) for depressive (IDS-SR) and anxiety (BAI) symptom severity across categories of food insecurity and financial strain. Estimated values were derived from prospective lagged multilevel linear regression analyses including repeated measures across 4 waves over 7 years. Percentages indicate the proportion of participants within each financial insecurity category. Model 2 adjusted for age, sex, education, number of household income contributors and comorbid baseline symptoms (i.e., BAI in models predicting IDS and vice versa). The right-hand plots show standardized beta-coefficients, representing standardized mean differences in symptom severity across financial insecurity categories. Significance of the linear trends across categories were tested using t-statistics.

Supplementary Figure 3. Prospective lagged multilevel linear regression analysis:

Depression / anxiety predicting food insecurity and financial strain at the next wave over a 7 year follow-up

Prospective analysis<br>Independent variables	No.	Model 1<br>Dependent variables	Model 2	p−value<br>for trend
**Depression**		**Experience of food insecurity**		
− Depressive disorder	3131	0.049 (0.015; 0.084)	0.051 (0.012; 0.089)	t=2.58; p=0.010
− Inventory of Depressive Symptomatology (IDS)	2921	0.065 (0.029; 0.102)	0.071 (0.013; 0.129)	t=2.38; p=0.02
**Anxiety**				
− Anxiety disorder	3131	0.029 (−0.005; 0.064)	0.019 (−0.019; 0.056)	t=0.96; p=0.34
− Beck Anxiety Inventory (BAI)	2915	0.059 (0.023; 0.095)	0.000 (−0.058; 0.058)	t=0.01; p=0.99
**Depression**		**Financial strain at end of month**		
− Depressive disorder	3131	0.029 (−0.005; 0.063)	0.028 (−0.010; 0.066)	t=1.43; p=0.15
− Inventory of Depressive Symptomatology (IDS)	2921	0.057 (0.021; 0.092)	0.081 (0.023; 0.139)	t=2.72; p=0.007
**Anxiety**				
− Anxiety disorder	3131	0.034 (0.000; 0.068)	0.029 (−0.009; 0.066)	t=1.51; p=0.13
− Beck Anxiety Inventory (BAI)	2915	0.041 (0.005; 0.076)	−0.024 (−0.082; 0.033)	t=−0.82; p=0.41

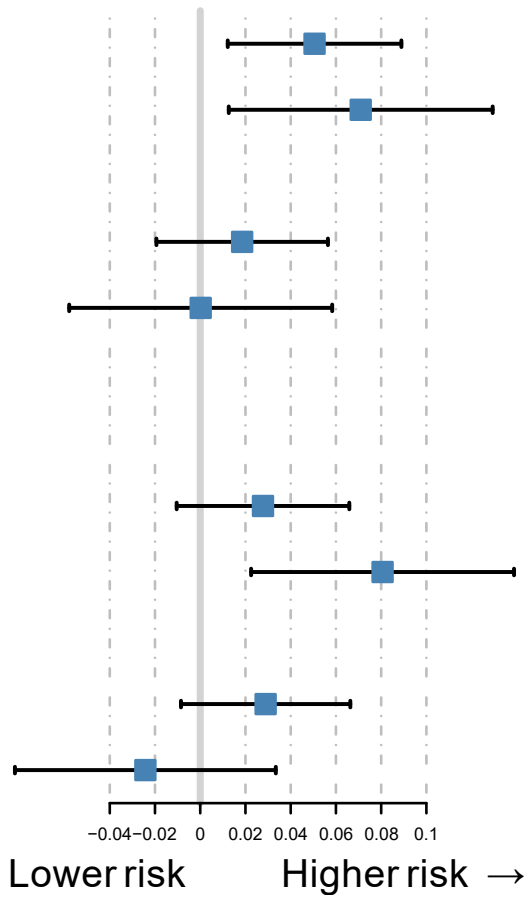

Note. Standardized regression coefficients (β) and 95% confidence intervals (CIs) (in parentheses) for financial insecurity outcomes (food insecurity and financial strain). Estimated values were derived from prospective lagged multilevel linear regression analyses including repeated measures across 4 waves over 7 years. Model 2 adjusted for age, sex, education, number of household income contributors, and comorbid baseline diagnoses and symptoms. The right-hand plots show standardized β-coefficients, representing associations between depressive and anxiety disorders and symptoms and subsequent levels of financial insecurity. Significance of these regression coefficients was tested using t-statistics.

Supplementary Figure 4. Prospective lagged multilevel logistic regression analysis:

Food insecurity and financial strain predicting depression / anxiety disorder at the next wave over a 13 year follow-up, limited to the low income group

Prospective analysis<br>Independent variables	Percentages	Model 1<br>Dependent variables	Model 2	p−value<br>for trend
**Experience of food insecurity**		**Depressive disorder**		
− Never	681 / 1687 (28.8%)	1.0 (ref.)	1.0 (ref.)	
− Occasionally	83 / 109 (43.2%)	1.52 (1.11−2.10)	1.41 (1.02−1.95)	
− Regularly/Often	61 / 59 (50.8%)	1.74 (1.18−2.58)	1.64 (1.10−2.45)	z=2.99; p=0.003
		**Anxiety disorder**		
− Never	459 / 1909 (19.4%)	1.0 (ref.)	1.0 (ref.)	
− Occasionally	50 / 142 (26.0%)	1.20 (0.83−1.73)	1.11 (0.77−1.61)	
− Regularly/Often	54 / 66 (45.0%)	2.31 (1.54−3.49)	2.18 (1.44−3.31)	z=3.39; p=0.001
**Financial strain at end of month**		**Depressive disorder**		
− Usually enough money	291 / 880 (24.9%)	1.0 (ref.)	1.0 (ref.)	
− Just enough money	387 / 771 (33.4%)	1.28 (1.06−1.56)	1.25 (1.03−1.52)	
− Not enough money	146 / 203 (41.8%)	1.55 (1.18−2.03)	1.46 (1.11−1.92)	z=2.93; p=0.003
		**Anxiety disorder**		
− Usually enough money	210 / 961 (17.9%)	1.0 (ref.)	1.0 (ref.)	
− Just enough money	246 / 912 (21.2%)	1.10 (0.88−1.38)	1.05 (0.84−1.32)	
− Not enough money	107 / 242 (30.7%)	1.48 (1.10−2.00)	1.37 (1.01−1.86)	z=1.80; p=0.07

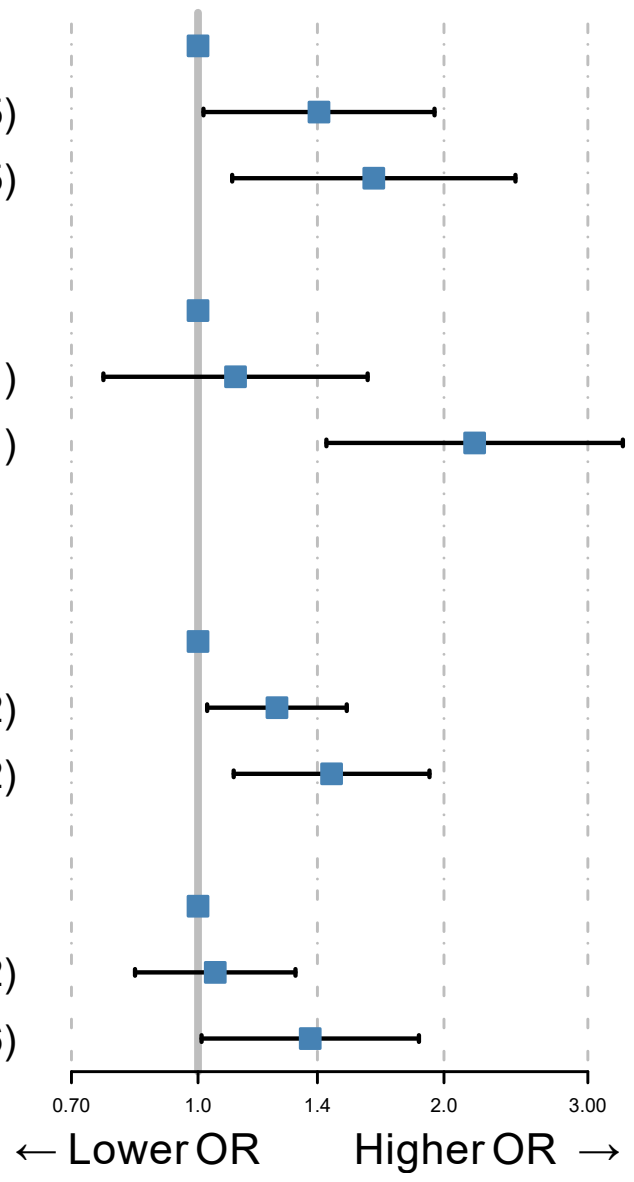

Note. Odds ratios (ORs)and 95% confidence intervals (CIs) (in parentheses) for current depressive and anxiety disorders (defined as an active diagnosis of any depressive or anxiety disorder in the past 6 months) across categories of food insecurity and financial strain. Estimated values were derived from prospective lagged multilevel logistic regression analyses including repeated measures across 4 waves over 13 years, restricted to observations at time points where participants reported a household income ≤ €2800. Percentages indicate the proportion of participants within each financial insecurity category. Model 2 adjusted for age, sex, education, number of household income contributors, and comorbid baseline diagnosis (i.e., anxiety disorder in models predicting depressive disorder, and vice versa). The right-hand plots show estimated ORs (with 95% CIs). Significance of the linear trends across categories were tested using z-statistics.

Supplementary Figure 5. Prospective lagged multilevel linear regression analysis:

Food insecurity and financial strain predicting depressive and anxiety symptom severity at the next wave over a 13 year follow-up, limited to the low income group

Prospective analysis<br>Independent variables	No.	Model 1<br>Dependent variables	Model 2	p−value<br>for trend
**Experience of food insecurity**		**Inventory of Depressive Symptoms (IDS)**		
− Never	2171 (89.2%)	17.45 (17.08−17.82)	17.39 (17.00−17.78)	
− Occasionally	167 (6.9%)	17.21 (15.87−18.56)	17.43 (16.07−18.79)	
− Regularly/Often	97 (4.0%)	19.67 (17.90−21.44)	19.64 (17.85−21.43)	t=5.32; p<0.001
		**Beck Anxiety Inventory (BAI)**		
− Never	2170 (89.1%)	9.27 (8.99−9.56)	9.17 (8.87−9.47)	
− Occasionally	168 (6.9%)	9.69 (8.66−10.72)	9.34 (8.31−10.38)	
− Regularly/Often	97 (4.0%)	11.76 (10.39−13.12)	11.36 (9.99−12.72)	t=5.29; p<0.001
**Financial strain at end of month**		**Inventory of Depressive Symptoms (IDS)**		
− Usually enough money	1082 (44.5%)	17.57 (17.04−18.10)	17.48 (16.94−18.02)	
− Just enough money	1045 (42.9%)	17.41 (16.88−17.95)	17.40 (16.84−17.95)	
− Not enough money	307 (12.6%)	17.64 (16.64−18.64)	17.71 (16.69−18.73)	t=3.18; p=0.002
		**Beck Anxiety Inventory (BAI)**		
− Usually enough money	1083 (44.5%)	9.21 (8.81−9.62)	9.23 (8.82−9.65)	
− Just enough money	1044 (42.9%)	9.37 (8.96−9.79)	9.16 (8.74−9.59)	
− Not enough money	307 (12.6%)	10.11 (9.34−10.88)	9.67 (8.89−10.45)	t=3.38; p=0.001

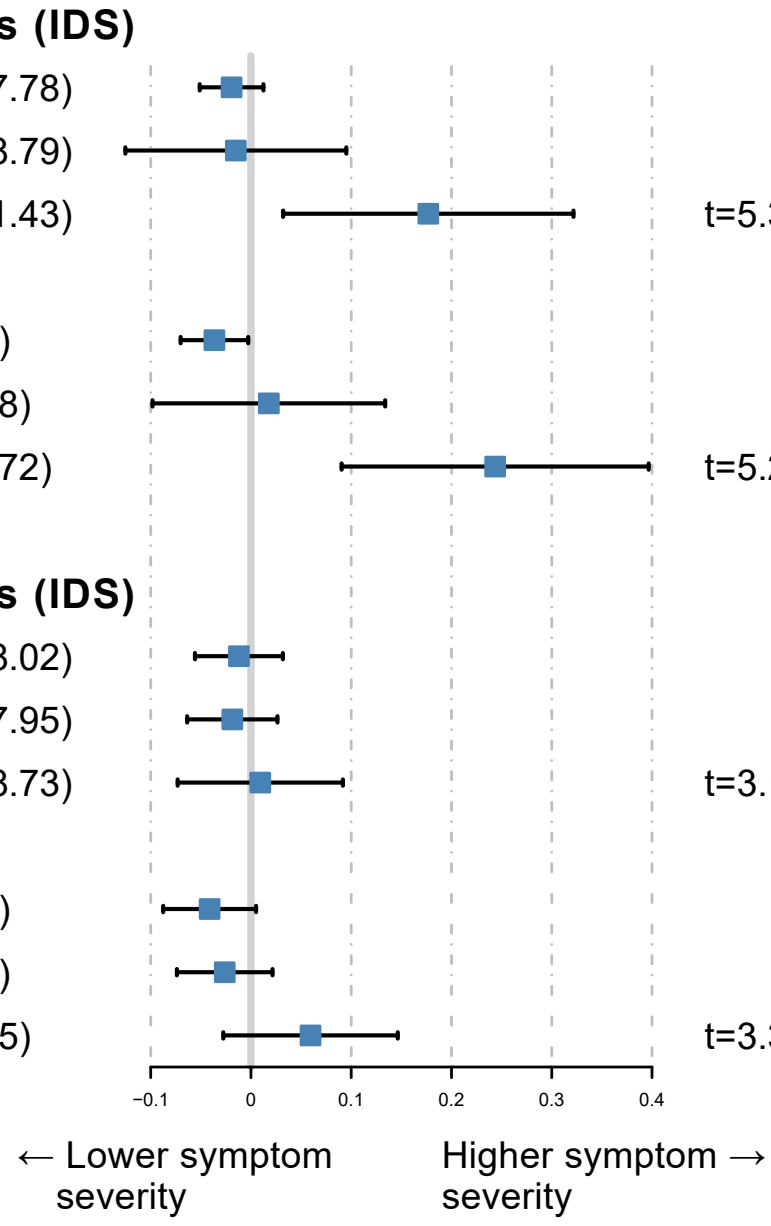

Note. Mean scores and 95% confidence intervals (CIs) (in parentheses) for depressive (IDS-SR) and anxiety (BAI) symptom severity across categories of food insecurity and financial strain. Estimated values were derived from prospective lagged multilevel linear regression analyses including repeated measures across 4 waves over 13 years, restricted to observations at time points where participants reported a household income ≤ €2800. Percentages indicate the proportion of participants within each financial insecurity category. Model 2 adjusted for age, sex, education, number of household income contributors and comorbid baseline symptoms (i.e., BAI in models predicting IDS and vice versa). The right-hand plots show standardized beta-coefficients, representing standardized mean differences in symptom severity across financial insecurity categories. Significance of the linear trends across categories were tested using t-statistics.

Supplementary Figure 6. Prospective lagged multilevel linear regression analysis:

Depression / anxiety predicting financial insecurity at the next wave over a 7 year follow-up, limited to the low income group

Prospective analysis<br>Independent variables	No.	Model 1<br>Dependent variables	Model 2	p−value
**Depression**		**Experience of food insecurity**		
− Depressive disorder	4743	0.043 (0.014; 0.072)	0.030 (0.000; 0.060)	t=1.94; p=0.053
− Inventory of Depressive Symptomatology (IDS)	4518	0.104 (0.075; 0.133)	0.103 (0.059; 0.147)	t=4.54; p<0.001
**Anxiety**				
− Anxiety disorder	4743	0.050 (0.022; 0.079)	0.040 (0.010; 0.070)	t=2.62; p=0.009
− Beck Anxiety Inventory (BAI)	4514	0.082 (0.054; 0.111)	0.003 (−0.041; 0.048)	t=0.15; p=0.88
**Depression**		**Financial strain at end of month**		
− Depressive disorder	4741	0.033 (0.004; 0.062)	0.016 (−0.015; 0.046)	t=1.02; p=0.31
− Inventory of Depressive Symptomatology (IDS)	4516	0.087 (0.059; 0.116)	0.081 (0.036; 0.125)	t=3.57; p<0.001
**Anxiety**				
− Anxiety disorder	4741	0.060 (0.032; 0.088)	0.052 (0.022; 0.081)	t=3.41; p=0.001
− Beck Anxiety Inventory (BAI)	4512	0.073 (0.045; 0.102)	0.010 (−0.034; 0.055)	t=0.46; p=0.64

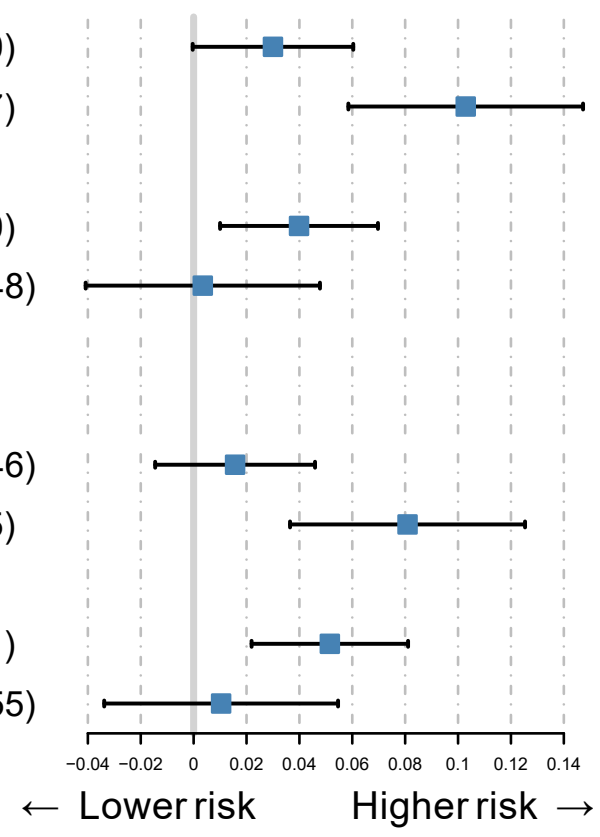

Note. Standardized regression coefficients (β) and 95% confidence intervals (CIs) (in parentheses) for financial insecurity outcomes (food insecurity and financial strain). Estimated values were derived from prospective lagged multilevel linear regression analyses including repeated measures across 4 waves over 13 years, restricted to observations at time points where participants reported a household income ≤ €2800. Model 2 adjusted for age, sex, education, number of household income contributors, and comorbid baseline diagnoses and symptoms. The right-hand plots show standardized β-coefficients, representing associations between depressive and anxiety disorders and symptoms and subsequent levels of financial insecurity. Significance of these regression coefficients was tested using t-statistics.

Supplementary Figure 7. Cross-sectional analyses:

Associations between financial insecurity and depressive / anxiety / worry symptoms severity

Cross−sectional analysis<br>Independent variables:	No.	Model 1<br>Dependent variables:	Model 2	p−value<br>for trend
**Experience of food insecurity**		**Inventory of Depressive Symptomatology (IDS)**		
− Never	5675 (95.3%)	14.69 (14.28−15.11)	14.89 (14.64−15.14)	
− Occasionally	178 (3.0%)	18.59 (17.31−19.87)	16.65 (15.61−17.70)	
− Regularly/Often	101 (1.7%)	22.73 (20.96−24.50)	17.41 (16.03−18.79)	t=4.5; p<0.001
		**Beck Anxiety Inventory (BAI)**		
− Never	5665 (95.3%)	7.73 (7.44−8.02)	7.84 (7.63−8.04)	
− Occasionally	179 (3.0%)	11.10 (10.15−12.05)	8.88 (8.03−9.73)	
− Regularly/Often	100 (1.7%)	13.06 (11.74−14.39)	9.52 (8.40−10.65)	t=3.5; p<0.001
		**Penn StateWorry Questionnaire (PSWQ)**		
− Never	3868 (94.5%)	25.85 (25.40−26.30)	25.87 (25.54−26.20)	
− Occasionally	139 (3.4%)	28.77 (27.33−30.21)	25.33 (24.12−26.54)	
− Regularly/Often	85 (2.1%)	31.11 (29.18−33.05)	25.56 (23.94−27.19)	t=−0.7; p=0.48
**Financial strain at end of month**		**Inventory of Depressive Symptomatology (IDS)**		
− Usually enough money	3972(66.7%)	14.06 (13.63−14.50)	14.64 (14.35−14.93)	
− Just enough money	1687 (28.3%)	16.24 (15.69−16.78)	15.54 (15.15−15.92)	
− Not enough money	293 (4.9%)	19.87 (18.82−20.93)	16.11 (15.29−16.94)	t=4.6; p<0.001
		**Beck Anxiety Inventory (BAI)**		
− Usually enough money	3967 (66.8%)	7.37 (7.05−7.68)	7.86 (7.62−8.10)	
− Just enough money	1685 (28.4%)	8.72 (8.32−9.11)	7.85 (7.54−8.16)	
− Not enough money	290 (4.9%)	11.16 (10.37−11.95)	8.75 (8.07−9.42)	t=1.6; p=0.12
		**Penn StateWorry Questionnaire (PSWQ)**		
− Usually enough money	2564 (62.7%)	25.27 (24.77−25.77)	25.76 (25.38−26.13)	
− Just enough money	1282 (31.3%)	26.85 (26.25−27.45)	25.87 (25.40−26.34)	
− Not enough money	245 (6.0%)	30.14 (28.98−31.29)	26.77 (25.80−27.74)	t=1.5; p=0.13

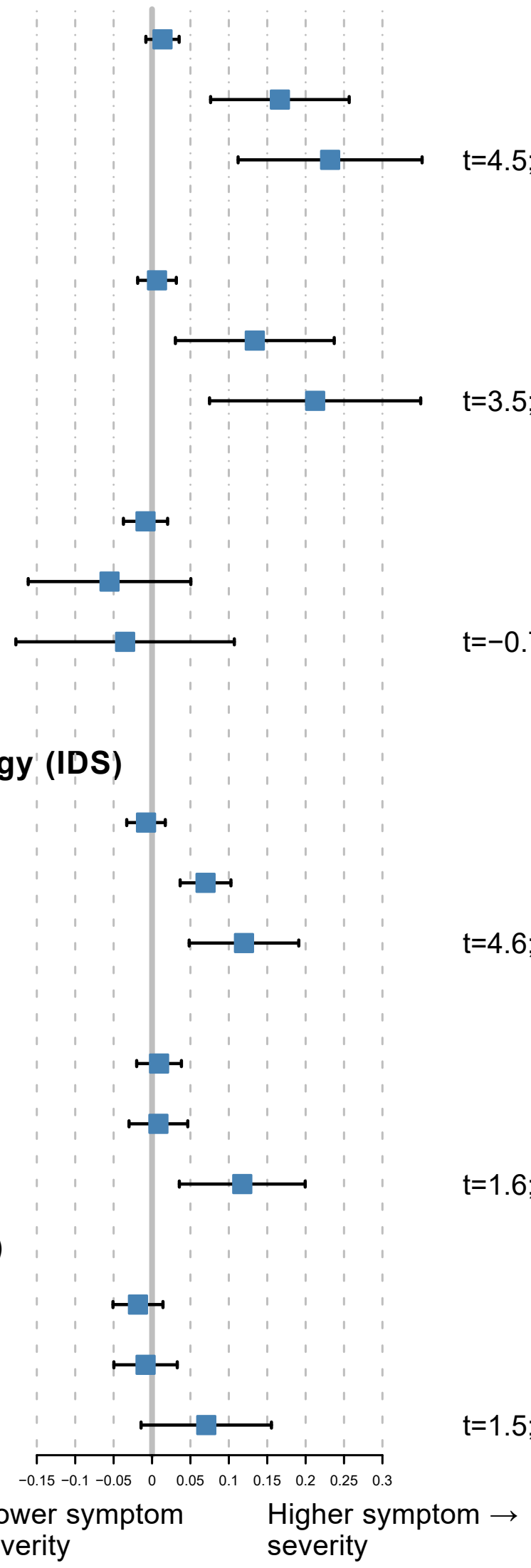

Note. Standardized regression coefficients (β) and 95% confidence intervals (CIs) (in parentheses) for depressive (IDS-SR), anxiety (BAI), and worry (PSWQ) symptom severity across categories of food insecurity and financial strain. Estimated values were derived from cross-sectional linear regression analyses at wave 6. Model 2 adjusted for age, sex, education, number of household income contributors and comorbid symptoms (i.e., BAI in models predicting IDS and vice versa).
